# A Novel MVA-Based Multiphasic Vaccine for Prevention or Treatment of Tuberculosis Induces Broad and Multifunctional Cell-Mediated Immunity in Mice and Primates

**DOI:** 10.1371/journal.pone.0143552

**Published:** 2015-11-24

**Authors:** Stéphane Leung-Theung-Long, Marie Gouanvic, Charles-Antoine Coupet, Aurélie Ray, Emmanuel Tupin, Nathalie Silvestre, Jean-Baptiste Marchand, Doris Schmitt, Chantal Hoffmann, Murielle Klein, Philip Seegren, Maria C. Huaman, Anthony D. Cristillo, Geneviève Inchauspé

**Affiliations:** 1 Department of Infectious Diseases, Transgene, Lyon, France; 2 Department of Smart Virus Lab, Transgene, Illkirch-Graffenstaden, France; 3 Department of Immunobiology, Advanced BioScience Laboratories, Inc., Rockville, Maryland, United States of America; Colorado State University, UNITED STATES

## Abstract

Bacille Calmette-Guérin (BCG) vaccination of new born babies can protect children against tuberculosis (TB), but fails to protect adults consistently against pulmonary TB underlying the urgent need to develop novel TB vaccines. Majority of first generation TB vaccine candidates have relied on a very limited number of antigens typically belonging to the active phase of infection. We have designed a multi-antigenic and multiphasic vaccine, based on the Modified Vaccinia Ankara virus (MVA). Up to fourteen antigens representative of the three phases of TB infection (active, latent and resuscitation) were inserted into MVA. Using three different strains of mouse (BALB/c, C57BL/6 and C3H/HeN), we show that a single vaccination results in induction of both CD4 and CD8 T cells, displaying capacity to produce multiple cytokines together with cytolytic activity targeting a large array of epitopes. As expected, dominance of responses was linked to the mouse haplotype although for a given haplotype, responses specific of at least one antigen per phase could always be detected. Vaccination of non-human primates with the 14 antigens MVA-TB candidate resulted in broad and potent cellular-based immunogenicity. The remarkable plasticity of MVA opens the road to development of a novel class of highly complex recombinant TB vaccines to be evaluated in both prophylactic and therapeutic settings.

## Introduction

In 2013, Tuberculosis (TB) claimed almost 1.5 million lives and the World Health Organization estimates that 9 million people developed the disease [1]. Control of TB is impeded by the strong increase in TB morbidity and mortality due to HIV co-infection, and the rise of multi-drug-resistant (MDR) and extensively drug-resistant (XDR) *Mycobacterium tuberculosis* (Mtb) strain. Full elimination of TB by 2050 can only be achieved with a better diagnosis, an effective vaccination strategy and a more effective treatment regimen. TB is primarily a pulmonary disease with an immune response dominated by a CD4/Th1/Th17 response early during the infection, and CD8 T cell responses increase as the infection progresses [2]. Only 5% of infected individuals develop an active form of the disease whereas for the remaining 95%, adaptive cellular immunity can contain the infection as latent tuberculosis infection (LTBI), during which bacteria survive in a non-replicating stage inside granulomas. A complete sterilization is rarely achieved and a long-term latent TB infection can give rise to reactivation. In low-endemic regions, reactivation of LTBI seems to be the main source of TB disease in the adult population whereas reinfection is more likely to happen in high burden areas [3, 4].

*Mycobacterium bovis* Bacillus Calmette-Guérin (BCG) is the only currently available vaccine against TB. However, its efficacy is suboptimal and it is not protective enough in adulthood [5]. There has been a concerted effort the last decade or more to develop new vaccine candidates. So far, none of the prophylactic candidates has resulted in pre-clinical models in sterilizing immunity or capacity to prevent the establishment of latent persistent tuberculosis infection. Post-exposure vaccination aiming at controlling reactivation and improving treatment is also an important component of the overall strategy being developed to control the global TB epidemic. Both development of more effective prophylactic vaccines as well as post-exposure vaccination are key strategies being pursued today [6].

The recent review by Da Costa *et al*. [7] while outlining the numerous platforms currently used in the effort towards developing novel recombinant TB vaccines (ranging from adjuvanted recombinant proteins to DNA and viral vectored vaccines) does highlight the limited number of antigens (Ag) entering the vaccine composition. First generation of vaccines contains basically one to two Ag, such as Ag85A, which are immunodominant antigens and belong to the active phase of infection [8–12]. More recently, actors in the field have started to introduce antigens from other phases of the infection such as latent antigens. Early results have shown that these second generation more “complexed” vaccines display enhanced efficacy, both in preventive and therapeutic studies [13–15]. The Modified Vaccinia Ankara (MVA) virus is one of the platforms used. It has shown encouraging results in mice, in particular when expressing more than 1 antigen [16] although a lead candidate expressing the Ag85A alone has failed to show protection in humans [10] in a BCG-booster situation in young infants, likely in great part because of its antigenic paucity.

In this study, taking advantage of the high plasticity as well as immunogenic and safety profile of the MVA vector [17, 18], we developed a recombinant MVA expressing 14 antigens representative of the three phases of TB infection (active, latent and resuscitation). As a pre-requisite to efficacy studies, we describe here its immunogenic profile both in mice and primates.

## Materials and Methods

### Mice

Six to eight weeks old female BALB/c, C57BL/6 or C3H/HeN mice were purchased from Charles River Laboratories (L’Arbresle, France) and housed at Plateau de Biologie Expérimentale de la Souris (PBES, Lyon, France) in ventilated cages.

The mouse experiments were carried out in accordance with the animal care guidelines of the European Union and French laws and were validated by the local Animal Ethic Evaluation Committee CECCAPP (Comité d’Evaluation Commun au Centre Léon Bérard, à l’Animalerie de transit de l’ENS, au PBES et au laboratoire P4) under the protocol number: ENS-2012-028.

### Housing and Care of Rhesus Macaques

The animals in this study were Indian rhesus macaques (*Macaca mulatta*) and were housed at the Advanced BioScience Laboratories, Inc. (ABL) animal facility. All animals were cared for and procedures performed under a protocol approved by the ABL Animal Care and Use Committee (animal welfare assurance no. A3467-01; protocol no. AUP571). Furthermore, the macaques in this study were managed according to the animal husbandry program of the ABL Animal Facility, which aims at providing consistent and excellent care to nonhuman primates at the vivarium. This program operates based on the laws, regulations, and guidelines promulgated by the United States Department of Agriculture (*e*.*g*., the Animal Welfare Act and its regulations, and the Animal Care Policy Manual), Institute for Laboratory Animal Research (*e*.*g*., Guide for the Care and Use of Laboratory Animals, 8th edition), Public Health Service, National Research Council, Centers for Disease Control, and the Association for Assessment and Accreditation of Laboratory Animal Care (AAALAC) International.

The nutritional plan utilized by the ABL Animal Facility consisted of twice daily feeding of Labdiet 5045 High Protein Primate Diet and food intake was closely monitored by Animal Research Technicians. This diet was also supplemented with a variety of fruits, vegetables, and other edible objects as part of the environmental enrichment program established by the Veterinary staff and enrichment Technician. Pairing of animals as part of the environmental enrichment program was managed by the enrichment technician. All primary enclosures and animal rooms were cleaned daily with water and sanitized at least once every two weeks. Viral challenges were performed under anesthesia (Ketamine administered at 10 mg/kg) and all efforts were made to minimize suffering. None of the animals were euthanized as part of this study.

ABL’s routine health surveillance consists of physical examinations, tuberculin tests as well as clinical tests and observations. Animals are periodically screened for viruses and pathogens such as STLV, SRV, measles and herpes B virus, and SIV using serological and sensitive real time PCR-based assays developed at ABL. Animal observations and general health checks will be performed twice daily, seven days a week, on all animals assigned to this program. Daily observations of all animals will be done to assess their health and well-being. Animals are observed for changes in stool condition, food consumption, evidence of trauma, signs of pain or distress, and the appearance of any clinical signs that indicate ill health. ABL has an established environmental enrichment plan for non-human primates compliant with the Animal Welfare Act. This enrichment plan includes social interaction through group housing, sensory and cognitive enrichment, and identification of and individualized treatment for psychological distress.

The program veterinarian is authorized to make decisions as to whether animals meet criteria that constitute humane endpoints that will result in removal of animals from study. The program veterinarian is in close communication with the principal investigator regarding the health status and medical conditions of animals enrolled in studies.

### Viruses

The MVA-based MVATG18377 virus encodes 14 Mtb antigens expressed as 3 protein fusions: RpfB-RpfD-Ag85B-TB10.4-ESAT-6, SF-Rv2029-Rv2626-Rv1733-Rv0111 and SR-Rv0569-Rv1813-Rv3407-Rv3478-Rv1807-TMR, where SF and SR are signal peptides of the F protein of measles virus and of the glycoprotein precursor of rabies virus ERA strain, respectively, and TMR is the membrane-anchoring peptide derived from the rabies glycoprotein. For some proteins, wild-type sequence (TB Database, www.tbdb.org) has been conserved, deleted or mutated. The first fusion, RpfB-RpfD-Ag85B-TB10.4-ESAT-6, was constituted by the residues 30 to 284 of RpfB fused to 54–147 fragment of mutated RpfD (E61K, T84A and Q113A), followed by the mature Ag85B polypeptide (40–325), and the full-length TB10.4 and ESAT-6 proteins. The second fusion, SF-Rv2029-Rv2626-Rv1733-Rv0111, was constituted by Rv2029 deleted of the last C-terminal 25 residues and mutated (D265N), followed by the full-length Rv2626 protein, the mature Rv1733 polypeptide (63–210) and Rv0111 deleted of all except one transmembrane domain (393–685). The SF signal peptide present at the N-terminus of the F protein of measles virus (Hallé strain, described in Genbank n° X05597-1) was positioned in-frame, 5’ to the Mtb fusion. The third fusion, SR-Rv0569-Rv1813-Rv3407-Rv3478-Rv1807-TMR, was constituted by the full-length Rv0569 protein, the mature Rv1813 polypeptide (35–143) followed by the full-length Rv3407, Rv3478 and Rv1807 proteins. The SR signal peptide present at the N-terminus of the glycoprotein precursor of rabies virus ERA strain (Genbank n° M38452) was positioned in-frame, 5’ to the Mtb fusion while the TMR membrane-anchoring peptide derived from the rabies glycoprotein (Genbank n°AY009097) was added in frame, 3’ to the Mtb fusion.

Synthetic genes coding for the different Mtb fusions were synthesized by Geneart (Regensburg, Germany). The sequences were optimized for human codon usage, a Kozak sequence (ACC) was added before the ATG starting codon and a 3’TTTTTNT transcriptional terminator sequence was added immediately after the stop codon.

The fusion SF-Rv2029-Rv2626-Rv1733-Rv0111 was placed under the control of the p7.5K promoter [19] while the fusion RpfB-RpfD-Ag85B-TB10.4-ESAT-6 was inserted downstream the pH5R promoter [20] and the fusion SR-Rv0569-Rv1813-Rv3407-Rv3478-Rv1807-TMR was inserted downstream the pB2R promoter. To create recombinant vaccinia virus, pTG17960 transfer vector allowing homologous recombination in the so-called deletion III corresponding site of the MVA genome was used [21]. The three expression fusions were cloned as head to tail concatemer into the pTG17960 transfer vector by standard cloning techniques. Recombinant MVA was then generated by standard procedures as described previously [21], by transfecting the relevant plasmid into MVA infected primary Chicken Embryo Fibroblast (CEF) and selecting plaques that were resistant to mycophenolic acid. The MVA strain, a subclone of MVA named MVATGN33.1, and its recombinant derivative were grown in CEF.

### Immunizations

Each mouse was injected subcutaneously with 1x10^7^ plaque-forming units (pfu) in 100 μL of Tris-HCl saccharose buffer. For ELISpot and intracellular cytokine staining (ICS) assays, MVA was injected once 7 days before the assay. For the cytotoxic T Lymphocyte (CTL) assay, MVA was injected twice 2 weeks apart and assay performed 7 days after last injection. For each immunological assay performed (see below), independent protocols were run.

The three rhesus macaques received MVATG18377 at Weeks 0, 8 and 18. All immunizations were given intramuscularly (i.m). A dose of 1x10^8^ pfu of recombinant poxvirus vector in 1.0 mL of Tris-HCl saccharose buffer was used in each immunization. At Weeks 0, 2, 10, 18, 20, 27, 29 and 31, peripheral blood mononuclear cells (PBMCs) were obtained from each immunized animal and Mtb antigen-specific T cellular immune responses were analyzed. Blood samples were processed following current procedures [22].

### Peptides and Antigens for *In Vitro* Stimulation

*In vitro* stimulations of splenocytes were performed using a synthetic peptide library (ProImmune, UK) constituted by 15-mer peptides overlapping by 11 residues. For each antigen, peptide pools were constituted containing up to 25 peptides. Therefore, depending on the length of a given antigen, 1 to 4 pools were required to cover its full sequence. An irrelevant peptide at 10 μM (GLL, located in NS3 protein of Hepatitis C Virus) was used as negative control in ELISpot assays. Pool of peptides covering HPV16 E7 sequence was used as irrelevant control for ICS assay.

### ELISpot assay

For mouse studies, IFNγ response was monitored one week after MVA injection. Six (BALB/c or C3H/HeN) or five (C57BL/6) mice per group were enrolled per experiment. IFNγ ELISpot assays were performed as previously described [23]. Briefly, splenocytes were stimulated *in vitro* with pools of peptides at 1 μM for 40 h in presence of IL2. Spots were quantified using an ELISpot microplate reader (CTL-Europe GmbH, Germany). Results are represented as mean value obtained for triplicate wells reported to 10^6^ cells. Threshold of positivity was defined as the mean value obtained in unstimulated condition plus 2 standard deviations (SD). Statistical analyses were performed using a Kruskal-Wallis test followed if significant (p<0.05) by a Mann-Whitney test.

For monkey study, Mtb antigen-specific IFNγ-secreting cells were measured in fresh PBMCs after stimulation with pools of peptides (15-mer overlapping by 11 amino acids) using ELISpot assay kits as described previously [24]. Assays were carried out in triplicate. After subtraction of spots in medium-only wells, the mean numbers of spot-forming cells (SFC) per million PBMCs were recorded.

### Triple Intracellular Cytokine Staining (ICS) Assay

Splenocytes of each mouse were collected 7 days after MVA injection (5 mice/group). Red blood cells were then lysed (Sigma). Spleen cells (2.10^6^/well) were seeded in flat-bottom 96-well plates and incubated at 37°C in αMEM culture medium (Gibco) supplemented with 10% FCS (PAA), 80 U/mL penicillin + 80 μg/mL streptomycin (PAN), 2 mM L-glutamine (Gibco), 1X non-essential amino acids (Gibco), 10 mM Hepes (Gibco), 1 mM sodium pyruvate (Gibco) and 50 μM β-mercaptoethanol (Gibco), in presence of 1 μM of all peptide pools for each antigen. GolgiPlug was added to splenocyte culture to block cytokine secretion during 5 h after 1 h stimulation with peptides, and cells were then stored at 4°C overnight.

After transfer into V-bottom 96-well plates, cells were washed with 1% FCS-PBS and incubated with 25 μL of anti-CD16/CD32 (clone 2.4G2, BD Biosciences) Fc Receptor block at a concentration of 2 μg/mL during 10 min at 4°C. Then, 25 μL of 1% FCS-PBS containing Live/Dead Violet (Invitrogen) and monoclonal antibodies against CD4 (rat anti-mouse CD4 APC-H7, clone GK 1.5, 0.5 μg/mL, BD Biosciences), CD8a (rat anti-mouse CD8a V500, clone 53–6.7, 2μg/mL, BD Biosciences) were incubated 30 min at 4°C. After washes, cells were fixed and permeabilized for 20 min in the dark at room temperature with Cytofix/Cytoperm and washed with Perm/Wash solution (BD Biosciences). After washes, 50 μL of Perm/Wash solution containing monoclonal antibodies against CD3 (hamster anti-mouse CD3-PerCP, clone 145-2C11, 2 μg/mL, BD Biosciences), IFNγ (rat anti-mouse IFNγ-A488, clone XMG1.2, 2 μg/mL, BD Biosciences), IL2 (rat anti-mouse IL2-PE, clone JES6-5H4, 2 μg/mL, BD Biosciences), TNFα (rat anti-mouse TNFα-APC, clone MP6-XT22, 0.5 μg/mL, BD Biosciences) were incubated 30 min at 4°C. After washes, cells were resuspended in 200 μL with 1% FCS-PBS and analyzed by flow cytometry using a BD FACS Canto II cytometer. A technical cut-off value was calculated as (25 / average number of CD3^+^CD4^+^ or CD3^+^CD8^+^ cells)*100. A response was then considered as positive if the percentage of cytokine-positive cell population was higher than the cut-off value.

### *In vivo* cytotoxic T lymphocyte (CTL) Assay

*In vivo* CTL assays were adapted from the protocol described by Fournillier and colleagues [25]. Briefly, peptide-pulsed splenocytes were labelled with different concentrations of carboxyfluorescein succinimidyl ester (CFSE, Molecular Probes) and/or CellTrace^™^ Violet (CTV, Molecular Probes). Six different labelled cell populations were mixed at an equal cell ratio and injected intravenously 7 days after the second immunization with either MVATGN33.1 or MVATG18377 (5 mice/group). After 20 h, splenocytes from recipient mice were analyzed by flow cytometry. Percentage of specific killing was determined by the following formula: % of specific lysis = [100 − (R_MVATG18377_/ R_MVATGN33.1_)] × 100 where R = number of peptide-pulsed target cells / number of unpulsed target cells. Statistical comparisons between groups immunized with MVATG18377 and MVATGN33.1 were performed using permutation resampling test applied on ratios (R_MVATG18377_ and R_MVATGN33.1_). *p* value less than or equal to 0.05 were considered as significant. Analyses were performed using R version 3.0.2.

## Results

### MVATG18377 Antigenic Design and *In Vitro* Characterization

Based on a scoring system taking into account antigen immunogenicity in both humans and animals, in addition to their efficacy in protection studies in animal models, we selected 14 Mtb Ag: 1. three well described antigens from the active phase, TB10.4 (Rv0288), ESAT-6 (Rv3875) and Ag85B (Rv1886) together with the Rv3478 belonging to the PE/PPE protein family [26, 27]; 2. eight latency-associated antigens: Rv1733, Rv2029, Rv0569, Rv0111, Rv2626, Rv1813, Rv3407 and the PE/PPE family Rv1807 protein (latent antigens have been associated with control of latent Mtb infection in both endemic and non-endemic countries [28]); 3. the resuscitation-promoting factors (Rpfs) RpfB and RpfD [29, 30].

**Fig 1:**
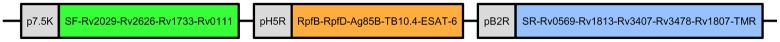
Schematic representation of the antigen fusions of MVATG18377. Three antigen fusions were inserted in the deletion III of MVA vector. The first fusion is constituted by the fusion of Rv2029, Rv2626, Rv1733 and Rv0111 proteins and is placed under the control of p7.5K promoter. The second fusion contains a fusion of RpfB-RpfD, Ag85B, TB10.4 and ESAT-6 proteins and its expression is driven by the pH5R promoter. The third fusion is constituted by the fusion of Rv0569, Rv1813, Rv3407, Rv3478 and Rv1807 proteins and is placed under the control of pB2R promoter. SF, signal peptide of the F protein of measles virus. SR, signal peptide of the glycoprotein precursor of rabies virus ERA strain. TMR, membrane-anchoring peptide derived from the rabies glycoprotein of PG strain.

These 14 antigens were combined in three expression fusions inserted in the MVA to generate the recombinant MVATG18377 (Fig 1). To insure a good protein expression and a high immunogenicity, organization of the fusions was the result of a design based on bioinformatics and biochemical analysis (search by homology with known structure, secondary structure prediction, hydrophobicity profile, naturally disordered region prediction, presence of transmembrane domain, signal peptide or known functional motifs) of each individual antigen. Expression of each fusion was monitored by Western blot analysis which showed detection of expected size polyproteins and/or dimeric forms of in *in vitro* MVA-infected cells (S1 Fig).

### The Multiphasic MVATG18377 Induces Broad IFNγ Response in mice

Although IFNγ is not recognized *per se* as being a correlate of protection in TB infection, it is believed to be an important contributor to control of mycobacterial replication [31]. ELISpot analysis was performed one week after mice immunization to investigate the specific IFNγ response induced by the MVATG18377. Immune response specific of each expressed Mtb antigen was evaluated in three different inbred mouse strains, BALB/c (H-2^d^), C57BL/6 (H-2^b^) and C3H/HeN (H-2^k^). Concerning large antigens (Rv2029, Rv2626, Rv1733, Rv0111, RpfB-RpfD, Ag85B, Rv3478 and Rv1807) for which sequence is covered by several pools of peptides, only results obtained with the pool leading to the highest response are shown. Data of all individual pools of peptides are shown in S2 Fig. Regardless of the mouse strain, a very strong IFNγ response specific of the MVA was detected in the control group as well as the MVATG18377-vaccinated group (> 800 spots/10^6^ cells, data not shown).

**Fig 2:**
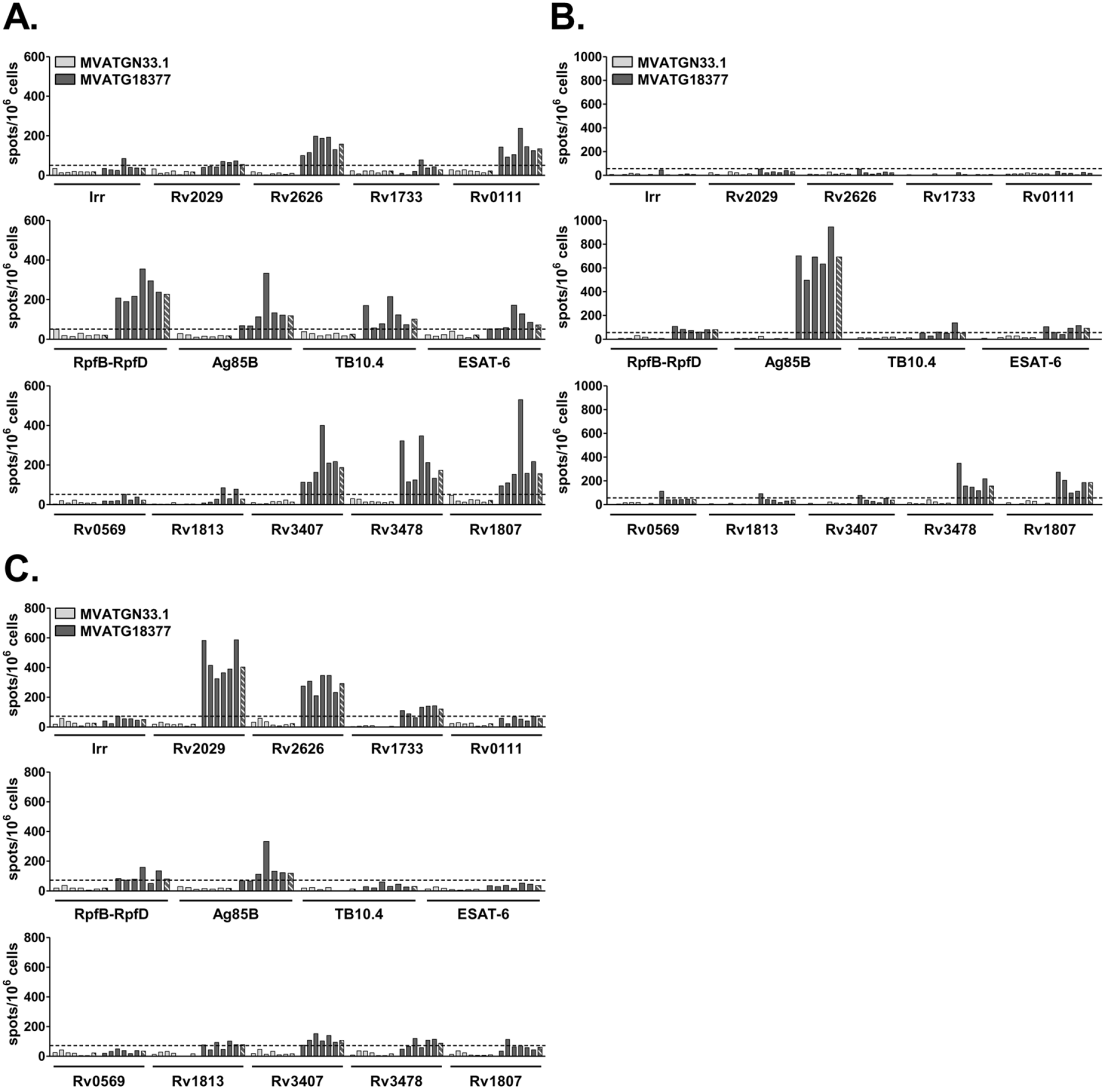
ELISpot analysis of IFNγ responses specific of MVATG18377-encoded Mtb antigens in different strains of mice. (**A**) BALB/c, (**B**) C57BL/6 and (**C**) C3H/HeN mice were immunized once with either MVATGN33.1 (light grey) or MVATG18377 (dark grey). Results are shown as the number of IFNγ-producing T cells (spots-forming cells) per 10^6^ splenocytes following stimulation with either peptide pools specific of each of the 14 antigens or the irrelevant GLL peptide (Irr). For long sequence antigens (Rv2029, Rv2626, Rv1733, Rv0111, RpfB-RpfD, Ag85B, Rv3478 and Rv1807), only results obtained with the peptide pool leading to the highest response are shown. Full bars represent individual mice and hatched bars represent median values of each group. The experimental cut-off value (dotted line) is represented for each mouse strain: 51 spots/10^6^ cells for BALB/c, 56 spots/10^6^ cells for C57BL/6 and 72 spots/10^6^ cells for C3H/HeN mice. Results are representative of two independent experiments.

In BALB/c mice, the 3 protein fusions were immunogenic and IFNγ-producing cells were detected for 11 out of 14 antigens (Fig 2A). In the first fusion, high IFNγ responses specific of Rv2626 and Rv0111 (158 and 134 spots/10^6^ cells, respectively) were observed while only a weak response or no response were observed for Rv2029 and Rv1733, respectively. All antigens of the second fusion were immunogenic. A strong IFNγ response (227 spots/10^6^ cells) specific of RpfB-RpfD was quantified along with responses ranging from 72 to 118 spots/10^6^ cells for Ag85B, TB10.4 and ESAT-6. Concerning the antigens present in the third fusion, while high IFNγ responses specific of Rv3407, Rv3478 and Rv1807 were measured (from 156 to 187 spots/10^6^ cells), no detectable response was observed for Rv0569 and Rv1813.

In C57BL/6 mice, IFNγ responses were detectable for half of the antigens expressed by the MVA (*i*.*e*. 7 out of 14) (Fig 2B). None of the latent antigens present in the first fusion was immunogenic. For the second fusion a very strong response to Ag85B (692 spots/10^6^ cells) and a weak one to ESAT-6 and RpfB-RpfD were detected. Among the antigens present in the third fusion, responses specific of Rv3478 and Rv1807 were quantified at 157 and 185 spots/10^6^ cells, respectively.

In the C3H/HeN mouse strain, IFNγ response was detectable for 9 out of 14 antigens (Fig 2C). IFNγ-producing cells (121 to 403 spots/10^6^ cells) specific of antigens present in the first fusion, Rv1733, Rv2626 and Rv2029, were detected. Overall, very weak IFNγ responses to some antigens of the two others fusions, RpfB-RpfD, Ag85B, Rv1813, Rv3407 and Rv3478 were measured.

Taken together, these results show that the multiphasic and poly-antigenic MVATG18377 vaccine candidate is a potent inducer of IFNγ response in mice. As expected dominance of the induced responses varies with the mouse haplotype and interestingly regardless of the mouse haplotype, IFNγ response is always detected for at least one antigen of each phase. The broadest immunogenicity pattern in terms of antigens targeted was found in BALB/c mice where 11 out 14 antigens are immunogenic.

### MVATG18377 Triggers Polyfunctional T Cells in Mice

**Fig 3:**
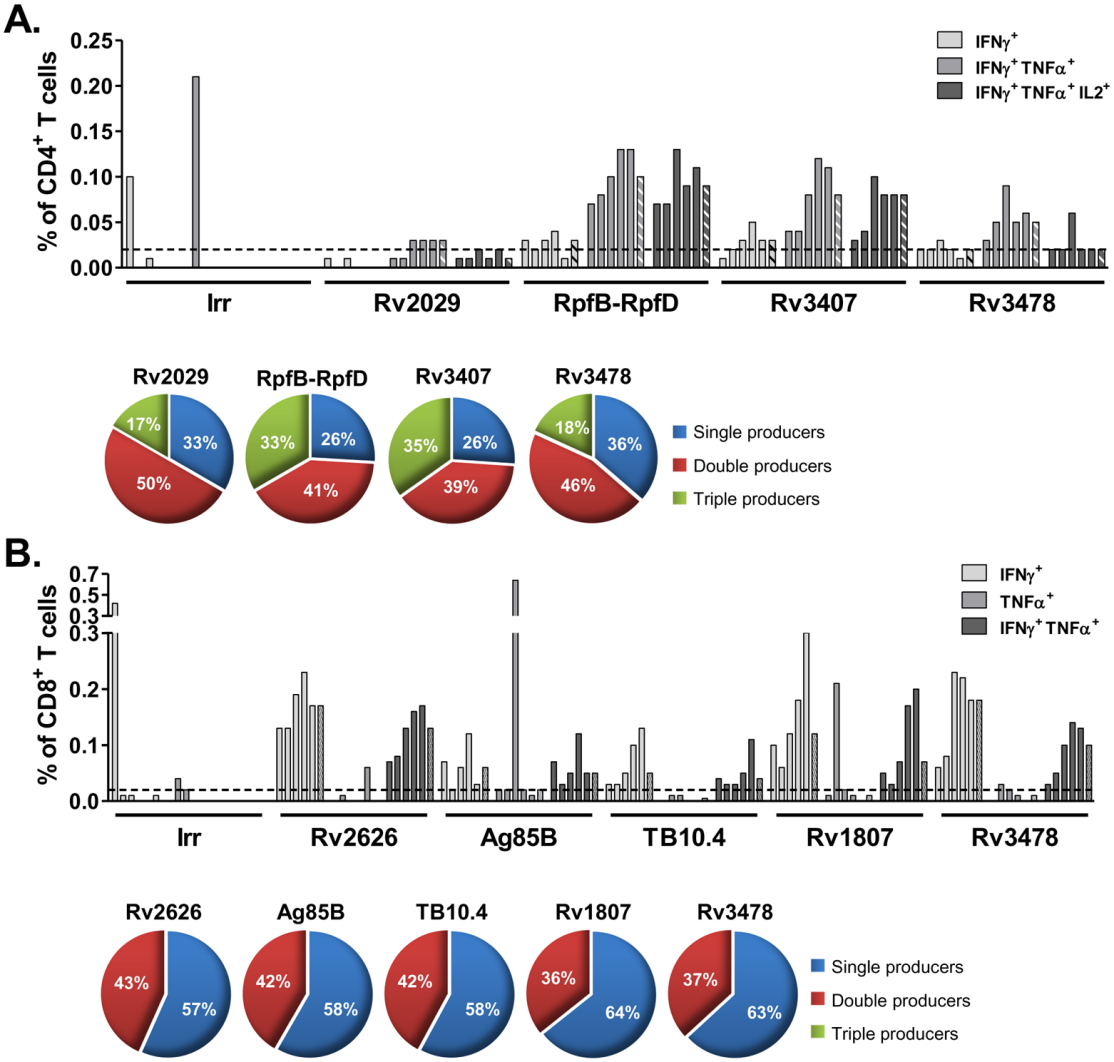
MVATG18377 induces multiple cytokines-producing T cells in BALB/c mice. Cells from MVATG18377-immunized BALB/c mice were stimulated with antigen peptide pools or an irrelevant E7 peptide (Irr) and IFNγ, IL2 and TNFα intracellular cytokine staining was measured by flow cytometry. Results are presented as (**A**) the percentage of IFNγ^+^, IFNγ^+^TNFα^+^ or IFNγ^+^TNFα^+^IL2^+^ cell subsets among total CD4 T cells or (**B**) percentage of IFNγ^+^, TNFα^+^ or IFNγ^+^TNFα^+^ cell subsets among total CD8 T cells. Plain bars represent response from individual mice and hatched bars represent median response for each cell subset. Cut-off value (dotted line, 0.02%) is represented for both CD4^+^ and CD8^+^ T cell responses. Only antigens with median values above the cut-off value are represented. For these antigens, only cell subgroups with a percentage above the cut-off value are represented. No response was detected in MVATGN33.1-immunized mice (data not shown). For each cell population, background signal obtained in unstimulated cell condition was subtracted. Pie charts represent a more global analysis for each responder antigen. All analyzed single (IFNγ^+^, TNFα^+^ and IL2^+^), double (IFNγ^+^TNFα^+^, IFNγ^+^IL2^+^ and TNFα^+^IL2^+^) or triple (IFNγ^+^TNFα^+^IL2^+^) cytokine producer cells are included under the corresponding color codes. Results are representative of two independent experiments.

To assess which functional T cell subsets contribute to the MVATG18377-induced response, we investigated co-expression of the Th1 cytokines, IFNγ, IL2 and TNFα, by ICS in vaccinated BALB/c and C57BL/6 mice. Regardless of the mouse strain, polyfunctional CD4^+^ and CD8^+^ T cells specific of the MVA vector were detected in the MVATGN33.1-vaccinated control group as well as the MVATG18377-vaccinated group (data not shown). In BALB/c mice, Th1 cytokine-expressing CD4^+^ T cells were elicited by 4 antigens out of 14: Rv2029, Rv3407, Rv3478 and the fusion RpfB-RpfD (Fig 3A). RpfB-RpfD-, Rv3407- and Rv3478-specific responses were composed of trifunctional (IFNγ^+^TNFα^+^IL2^+^), bifunctional (IFNγ^+^TNFα^+^) and monofunctional (IFNγ^+^) CD4^+^ T cell subsets. Rv2029 elicited only a very weak frequency (0.03%) of bifunctional (IFNγ^+^TNFα^+^) CD4^+^ T cells. Frequencies of Rv3478-specific CD4^+^ T cells that significantly exceeded the threshold comprised mostly IFNγ^+^TNFα^+^ cells (0.02 to 0.05% of CD4^+^ T cells). RpfB-RpfD and Rv3407 specific responses were predominantly comprised of IFNγ^+^TNFα^+^IL2^+^ (0.09% and 0.08%, respectively) and IFNγ^+^TNFα^+^ (0.10% and 0.08%, respectively). When considering only the cytokine-positive cell populations, it clearly appears that polyfunctional CD4^+^ T cells specific of RpfB-RpfD and Rv3407 represent a high proportion of this population (33% and 35%, respectively).

As shown in Fig 3B, in BALB/c mice, MVATG18377 induced cytokines-producing CD8^+^ T cells specific of antigens of latent phase, Rv2626 and Rv1807, and active phase, Rv3478, Ag85B and TB10.4. The CD8^+^ T cell cytokine response was predominantly composed of IFNγ^+^ and IFNγ^+^TNFα^+^ CD8^+^ T cells. Frequencies of bifunctional (IFNγ^+^TNFα^+^) CD8^+^ T cell specific of Rv2626, Rv1807 and Rv3478 ranged from 0.10 to 0.18%. For both active antigens, TB10.4 and Ag85B, lower frequencies of bifunctional CD8^+^ T cells (0.04 to 0.06%) were detected. In contrast to CD4^+^ T cells, no triple producers CD8^+^ T cells were detected. Regardless of the antigen, about 60% of the cytokine-expressing CD8^+^ T cells were single positive for IFNγ and 40% co-produced IFNγ and TNFα.

Overall these results are in accordance with ELISpot data. Among the 11 ELISpot-positive antigens observed in BALB/c mice, IFNγ-producing CD4^+^ and CD8^+^ T cells (single and multiple producers) specific of 9 of these antigens were identified by ICS assay.

**Fig 4:**
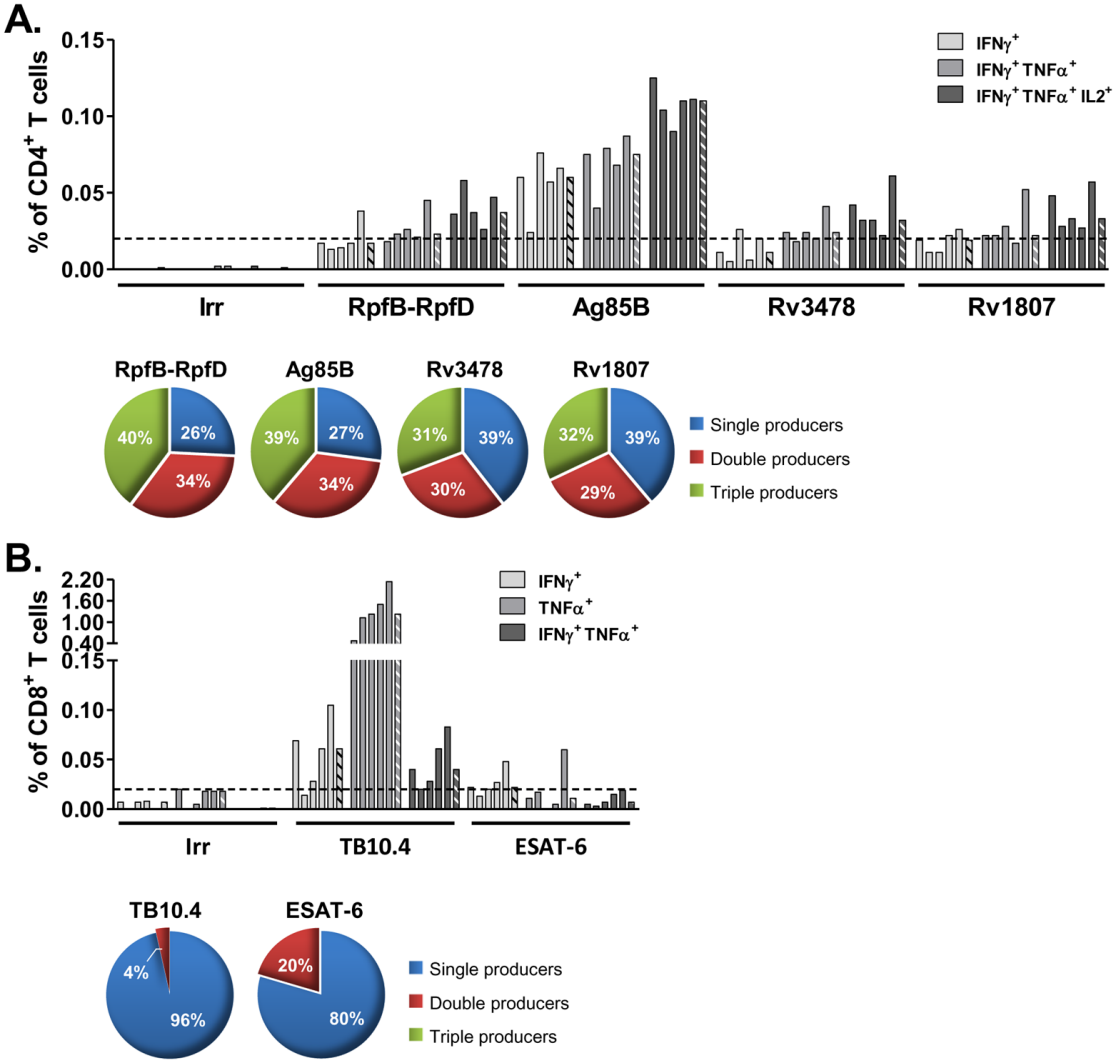
MVATG18377 induces multiple cytokines-producing T cells in C57BL/6 mice. Cells from MVATG18377-immunized C57BL/6 mice were stimulated with antigen peptide pools or an irrelevant E7 peptide (Irr). Results are presented as (**A**) the percentage of IFNγ^+^, IFNγ^+^TNFα^+^ or IFNγ^+^TNFα^+^IL2^+^ cell subsets among total CD4 T cells or (**B**) percentage of IFNγ^+^, TNFα^+^ or IFNγ^+^TNFα^+^ cell subsets among total CD8 T cells. Plain bars represent response from individual mice and hatched bars represent median response for each cell subset. Cut-off value (dotted line, 0.02%) is represented for both CD4^+^ and CD8^+^ T cell responses. Only antigens with median value above the cut-off value are represented. For these antigens, only cell subgroups with a percentage above the cut-off value are represented. No response was detected in MVATGN33.1-immunized mice (data not shown). For each cell population, background signal obtained in unstimulated cell condition was subtracted. Pie charts represent a more global analysis for each responder antigen. All analyzed single (IFNγ^+^, TNFα^+^ and IL2^+^), double (IFNγ^+^TNFα^+^, IFNγ^+^IL2^+^ and TNFα^+^IL2^+^) or triple (IFNγ^+^TNFα^+^IL2^+^) cytokine producer cells are included under the corresponding color codes. Results are representative of two independent experiments.

In C57BL/6 mice, Th1 cytokine-expressing CD4^+^ T cells were observed following stimulation with RpfB-RpfD, Ag85B, Rv3478 and Rv1807 peptides (Fig 4A). A low proportion of cytokine-positive CD4^+^ T cells was detectable for RpfB-RpfD, Rv3478 and Rv1807 (0.02 to 0.04%). Stimulation with Ag85B peptides revealed higher proportions of specific CD4^+^ T cells with 0.11% of IFNγ^+^TNFα^+^IL2^+^ and 0.08% of IFNγ^+^TNFα^+^ cell subsets, which is consistent with the IFNγ ELISpot data (Fig 2B).

Regarding the CD8^+^ T cell response, only 2 antigens belonging to the active phase, ESAT-6 and TB10.4, displayed detectable cytokine-producing cells (Fig 4B). ESAT-6 specific response was composed of a low frequency of IFNγ^+^ CD8^+^ T cells (0.02%). TB10.4 specific response was composed of low but significant frequencies of IFNγ^+^ and IFNγ^+^TNFα^+^ CD8^+^ T cells (0.06% and 0.04%, respectively) as well as a high proportion of TNFα^+^ CD8^+^ T cells (1.23%). TB10.4-specific IFNγ^+^ cell subsets were unexpected as no IFNγ response was detected by ELISpot assay (Fig 2B). As in BALB/c mice, in C57BL/6 mice no triple cytokine CD8^+^ T cell population was detected. The distribution of cell subsets showed that single cytokine producers constitute the main population (*e*.*g*. up to 96% in case of TB10.4).

In conclusion, these results reinforce the data obtained by ELISpot assay. In fact the seven antigens those giving a positive response in IFNγ ELISpot assays (Fig 2B) were also shown to be positive for IFNγ staining in ICS.

### Mtb Antigen-Specific CD8 T Cells Induced by MVATG18377 Are Cytolytic *In Vivo*

**Fig 5:**
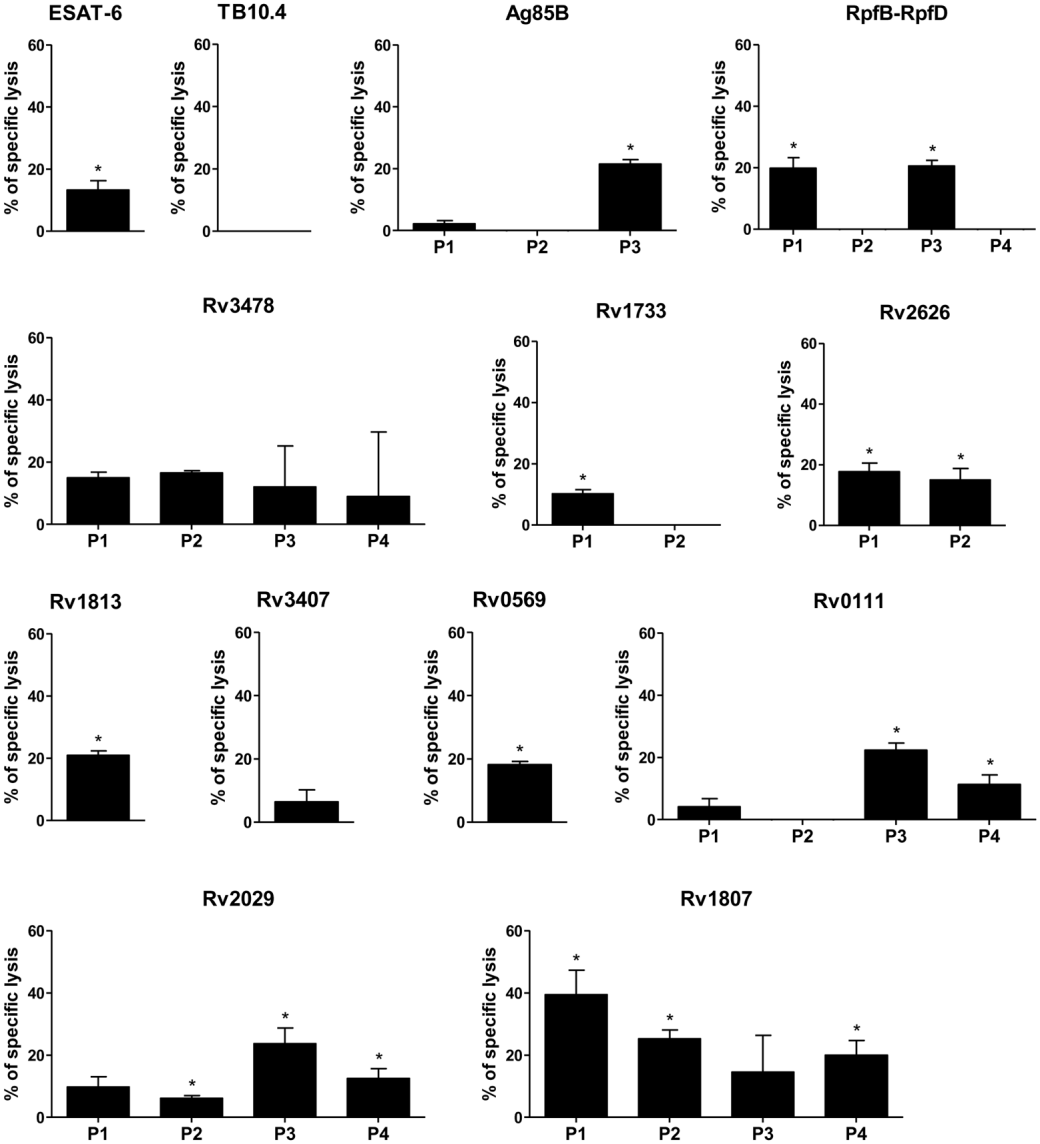
*In vivo* CTL analysis in immunized BALB/c mice. Mice were immunized twice with either MVATGN33.1 or MVATG18377. Labelled target cell populations pulsed with individual antigen peptide pool were adoptively transferred into those 7 days after last immunization. Results are expressed as mean ± S.D. of the percentage of specific killing based on the relative ratio of target cells present in MVATG18377-vaccinated mice compared to those in the MVATGN33.1-vaccinated mice. For each time point and antigen peptide pool (P), significant lysis is indicated by * (p<0.05) using a permutation resampling test.

We investigated whether the CD8^+^ T cells elicited following MVATG18377 vaccination display cytotoxicity *in vivo*. *In vivo* CTL assays were performed in BALB/c mice immunized twice with vaccine candidate using target cells pulsed with pool of antigen-specific peptides. We first analyzed the kinetics of the CTL response by adoptively transferring target cells at different times after the last immunization (3, 5 or 7 days after the last immunization). The broadest CTL killing response in terms of number of antigens was observed 7 days after the last immunization (data not shown). This time point was selected in the next experiments. Mice immunized with either the MVATG18377 or the control vector generated a strong CTL killing response to the MVA peptide-pulsed targets (80–90%, data not shown). Interestingly, MVATG18377-immunized mice developed a CTL killing response specific of 11 out of 14 antigens expressed by the virus (Fig 5). CTL responses targeting cells pulsed with Ag85B, RpfB-RpfD, Rv1813, Rv0111, and Rv2029 peptides displayed a percentage of lysis ranging from 20 to 25%. ESAT-6- and Rv2626-pulsed target cells were killed less efficiently with a percentage of lysis inferior to 20% whereas Rv1807-pulsed target cells displayed the highest percentage of lysis of 39% (with peptide pool 1). Surprisingly, while no IFNγ response and no cytokine response specific of Rv0569 and Rv1733 could be detected in BALB/c mice (Figs 2A and 3), MVATG18377 triggered significant cytotoxicity directed against both antigens (18% and 10%, respectively). In contrast, no significant specific cytotoxic activity was detected for three antigens, *i*.*e*. TB10.4, Rv3478 and Rv3407, under these experimental conditions.

### The Multiphasic MVATG18377 Induces High IFNγ Response in Non-Human Primates

**Fig 6:**
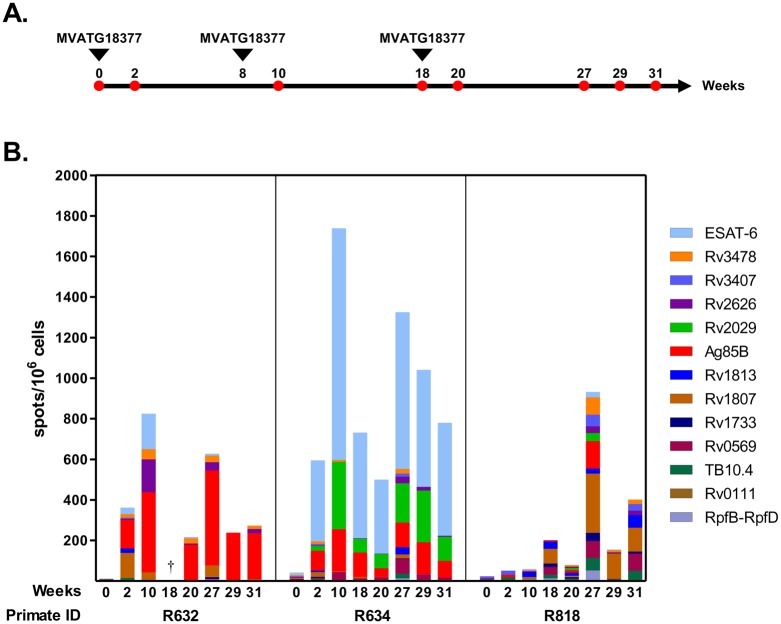
ELISpot analysis of IFNγ responses specific of MVATG18377-encoded Mtb antigens in non-human primates. (**A**) Overview of primate experimental design. Three primates were immunized thrice with MVATG18377 at Weeks 0, 8 and 18 (arrowhead). IFNγ ELISpot assays were performed with PBMCs collected at indicated time points (red point). (**B**) Antigen-specific IFNγ responses. Results are shown as the number of IFNγ-producing cells (spots) per 10^6^ cells following stimulation with peptide pools specific of each of the 14 antigens. At each time point, cumulative results following stimulation with each of 14 antigens are represented. A color code is used for each antigen. †, data are not available due to cell culture issues.

Non-human primates (NHP) are well described as a valuable animal model for studying TB as the disease is closely similar to that occurring in humans [32, 33] hence reinforcing value in the testing of TB vaccine candidates, at least in prophylaxis [34–36]. Immunogenicity of the MVATG18377 was evaluated in this model by investigating induction of specific IFNγ responses. Three NHPs were immunized thrice at Weeks 0, 8 and 18 and immune response was determined by IFNγ ELISpot assays using PBMCs collected at different time points along the study (Fig 6A).

As illustrated in Fig 6B, all monkeys were TB-naive before starting the study as no Mtb antigen-specific IFNγ production was detected at Week 0. Interestingly, IFNγ-producing cells were detected in 2 out of 3 NHPs following the first vaccine injection. In R632 monkey, responses specific of Ag85B and Rv1807 were indeed measured (142 and 123 spots/10^6^ cells, respectively). In addition to Ag85B, ESAT-6-specific significant IFNγ response was detected in R634 monkey (98 and 400 spots/10^6^ cells, respectively).

At Week 10, *i*.*e*. two weeks after the boost vaccination, specific responses continued to be measured in both R632 and R634 NHPs. Total IFNγ levels increased 2 and 3 times in R632 and R634 respectively, when compared to responses at Week 2. While a boost effect was clearly observed for Ag85B, the signal specific of Rv1807 did not increase, and tended to diminish in R632. Surprisingly, in this animal the second vaccine injection led to induction of IFNγ-producing cells specific of both ESAT-6 and Rv2626 at high level, and of Rv3478 although to a lesser extent. A boost effect was also observed in R634. A 3-fold increase of the number of spots was indeed counted in response to ESAT-6 peptides (from 400 to 1143 spots/10^6^ cells at Week 2 and Week 10, respectively). Likewise, Ag85B-specific response doubled after the boost. In addition, the second MVA injection gave rise to IFNγ responses specific of both latent Rv2029 and Rv0569 antigens (333 and 42 spots/10^6^ cells, respectively).

Immune response was further evaluated 8 weeks after the boost injection, *i*.*e*. at Week 18. At this time point, no data for R632 primate was generated due to low cell responsiveness. While overall IFNγ levels decreased in R634 monkey, weak signal increase was measured for Rv1813, Rv1807 and Rv0569 in the third macaque R818. Unexpectedly, two weeks after the third vaccination, *i*.*e*. at Week 20, all IFNγ responses were lower than that observed at Week 18 and even after the first boost (Week 10).

Long lasting specific response was also investigated and IFNγ ELISpot assays were carried out at Weeks 27, 29 and 31, *i*.*e*. from 7 weeks after the last MVA injection. Whereas only Ag85B-specific response was maintained at these late time points in R632 macaque, three antigen-specific responses (ESAT-6, Rv2029 and Ag85B) were still measured at high levels in R634 macaque. Strikingly, the highest and broadest IFNγ responses were detected at Week 27 in R818 monkey. IFNγ-producing cells specific of antigens belonging to the three phases of disease, Rv1807, Rv3407, Rv3478, Rv0569, Ag85B, TB10.4 and RpfB-RpfD were indeed revealed following *in vitro* stimulation. However, at Week 31 even if signals were lower, IFNγ-producing cells specific of Rv1813, Rv1807, Rv0569 and TB10.4 were still detected.

Taken together, these results demonstrate that the multiphasic and multi-antigenic MVATG18377 vaccine candidate is also a potent IFNγ inducer in non-human primates in addition to mice. Dominance of the antigenic responses varies with the monkey. As in mice, the different antigenic patterns might be related to the monkey haplotype.

## Discussion

Contrary to the dogma that prevailed for a long time, there is today a consensus on expression of Mtb antigens being rather continuous, independent of the antigen nature, than limited to a specific phase of infection [37]. For example, although with variable intensity, so called “Rv2660” can be found during latency as well as active phase [13]. In order to best capture this feature and optimize vaccine design, we have constructed a novel vaccine, the recombinant MVATG18377, that contains three protein fusions encompassing 14 Mtb antigens belonging to the three phases of the TB disease, *i*.*e*. active, latent and resuscitation. We show here using three different lines of mice that immunization with MVATG18377 induces T cell-based immune responses specific of all 14 encoded Mtb antigens. In particular, we were able to demonstrate that these responses involved both CD4^+^ and CD8^+^ T cells. One single injection of MVATG18377 was sufficient to induce Mtb antigen-specific CD4 T cells that were capable to secrete IFNγ but also multiple cytokines (polyfunctional cells), *i*.*e*. IFNγ^+^TNFα^+^ double producers but also IFNγ^+^TNFα^+^IL2^+^ triple producers. Polyfunctional T cells induced by vaccines have been shown to play a key role in protection against Mtb infection in mice [38]. Beside Th1 CD4^+^ T cells, CD8^+^ T cells have also been more recently described as important components for protection [39]. CD8^+^ T cells are recruited to the lung during Mtb infection and are found in the granulomas of infected people. Studies have shown that CD8^+^ T cells contribute to overall immunity to TB by the release of cytokines and antimicrobial peptides (perforin and granulysin) that promote killing of infected macrophages and intracellular mycobacteria [40]. Our data show that MVATG18377 is able to induce Mtb antigen-specific CD8^+^ T cells with a distinct cytokine profile compared to the CD4^+^ T cells one. They mainly produce single cytokines (IFNγ or TNFα) and are capable to produce both IFNγ and TNFα. In addition, we show that these CD8^+^ T cells are able to kill naive splenocytes loaded with peptides of 11 out 14 of the MVA-expressed Mtb antigens which may confer an enhanced protection against Mtb [41].

The multiple readouts used in the current study highlight that each antigen present in the vaccine has a specific profile of T cell population (Table 1). Rv1807-specific CD8^+^ T cells presented a high CTL activity and were double positive for IFNγ and TNFα. However, we did not find a systematic correlation between CTL activity and CD8^+^ T cell polyfunctionality as double cytokine producers TB10.4 and Rv3478 specific CD8^+^ T cells for example did not display any detectable CTL activity. Rv3478, RpfB-RpfD and Rv3407 elicited a very robust IFNγ response as well as polyfunctional CD4^+^ T cells. However a robust IFNγ response was neither required nor sufficient to induce a polyfunctional CD4^+^ response. Finally, Rv1807 elicited a good IFNγ response without polyfunctionality whereas Rv2029 elicited polyfunctional CD4^+^ T cells with a rather weak IFNγ response. Each antigen present in the vaccine candidate induces T cell population unique regarding their cytokine profile and function.

**Table 1:** Summary of immune responses induced by the 14 Mtb antigens-expressing MVATG18377 vaccine candidate in BALB/c mice.

	Active				R	Latent							
	Ag85B	ESAT-6	TB10.4	Rv3478	RpfB-RpfD	Rv1813	Rv2029	Rv0111	Rv0569	Rv1733	Rv1807	Rv2626	Rv3407
**Triple producer CD4 T cells**	-	-	-	✓	✓	-	✓	-	-	-	-	-	✓
**Double producer CD8 T cells**	✓	-	✓	✓	-	-	-	-	-	-	✓	✓	-
**IFNγ ELISpot**	++	+	+	+++	+++	-	+	++	-	-	+++	++	+++
**CTL**	++	+	-	-	++	++	++	++	+	+	+++	+	-

R, Resuscitation.

Ag-specific triple producer CD4 T cells and double producer CD8 T cells are indicated by a check mark. IFNγ ELISpot: group median value of spots detected using the following ranking:

-, median<cut-off;

+, 1x cut-off < median < 2x cut-off;

++, 2x cut-off < median < 3x cut-off;

+++, median > 3x cut-off.

CTL: -, no activity;

+, CTL < 20%;

++, 20% < CTL < 30%;

+++, CTL > 30%).

We show here that the MVATG18377 is immunogenic in monkeys. As in mice, no major immunodominance was observed that is a very encouraging feature for a vaccine candidate. Although antigen specific IFNγ responses could be detected as early as following a prime immunization, additional doses appear beneficial to both increase and sustain the levels of antigen T cell responses. Not surprisingly, immunogenic pattern was different between macaques likely reflecting the diversity of MHC haplotypes in these monkeys. Such diversity is expected to be seen in humans and design of the MVATG18377 that includes up to 14 antigens from all phases of infection should insure the mounting of an immune response specific of a minimum of antigens representative of all 3 phases of infection.

Our data show that the MVATG18377 candidate vaccine is highly immunogenic both in mice and non-human primates using T cell-based immuno-assays. In addition to the production of the three cytokines, IFNγ, TNFα and IL2, assessed in the current study it would be worthwhile to investigate whether other cytokines, such as those belonging to the IL17 family and known to play important role in control of TB infection [42–44], are also induced by MVATG18377 candidate.

The last decade has witnessed the development of novel TB vaccine candidates with more than a dozen undergoing clinical assessment. Subunit vaccines aim to induce immune responses to a single or a few antigens selected based on their immunogenicity during infection [45]. Two main protein-based vaccines composed of different sets of antigens covering two phases of disease (active and latent) have shown protective efficacy in pre-clinical studies and are currently in clinical trials. The IC31-adjuvanted Hybrid 56 (H56) is a fusion of Ag85B, ESAT-6 and Rv2660c [13, 34], and ID93 adjuvanted with GLA-SE combines a novel set of antigens from both active and latent phases, Rv2608, Rv3619, Rv3620 and Rv1813 [46, 47]. These different candidates demonstrate that combining multiple antigens in one vaccine leads to increased vaccine efficacy against Mtb. The MVA platform has been used in the development of a first generation vaccine with the specific MVA85A candidate. This vaccine, used as a BCG booster prophylactic vaccine in young infants, has reached phase 2b. Despite promising immunogenicity, MVA85A has failed to show evidence of protection against Mtb infection [10]. Mechanism of failure of protection is not known but hypotheses include a too low antigen complexity (only one antigen), use of a kinetic of vaccine administration too close to the BCG priming (boosting effect null or suboptimal), challenge to mount a strong immunogenicity with the used dose, schedule and route in young infants, limited functionality of induced T cells. Clearly, the MVATG18377 developed here provides for a much larger antigenic diversity and as shown polyfunctionality of induced T cell responses. In addition, its use by the aerosol route, recently shown as a promising route for MVA-based TB candidates [48] may result in optimal efficacy. Another interesting positioning for the MVATG18377 would be that of a primer or booster of adenovirus-based vaccines. Recent encouraging results in a number of infectious diseases indications (*e*.*g*. Ebola [49], Malaria [50]) cannot be ignored and are worth exploring in the TB field.

In conclusion, the MVATG18377 vaccine constitutes the most complex virus-based vaccine developed in the TB field today. Beside the importance of developing a novel preventive vaccine, one additional challenge to improve fight against tuberculosis is to set up novel strategies aiming at improving treatment outcomes and shortening treatment duration of drug-resistant disease together with preventing rebound of the disease and/or reinfection following treatment. Combination of an active-targeted immunotherapy, such as provided by the multiphasic MVATG18377, with antibiotherapy is an attractive approach that should also be considered in particular as MVA-based immunotherapeutics have already shown encouraging clinical results in oncology and chronic viral infections [17].

## Supporting Information

S1 Fig Expression of the Mtb fusion proteins in A549 cells infected with MVATG18377. A549 cells were infected or not (mock) with MVATG18377 or MVATGN33.1 and cell extracts analyzed by Western blot. Rv2029-Rv2626-Rv1733-Rv0111 fusion (*, expected molecular weight: 98.4 kDa) was detected using (**A**) mouse monoclonal anti-Rv2626 26A11 antibody or (**B**) rabbit polyclonal anti-Rv0111 serum. Major proteolytic products were observed with a Rv2626-specific antibody (around 70.0 kDa) and with Rv0111-specific serum (around 30.0 kDa), suggesting a proteolytic cleavage of this fusion. (**C**) RpfB-RpfD-Ag85B-TB10.4-ESAT-6 fusion (*, expected molecular weight: 87.0 kDa) was detected using mouse monoclonal anti-ESAT-6 HYB076-08 antibody and (**D**) Rv0569-Rv1813-Rv3407-Rv3478-Rv1807 fusion (*, expected molecular weight: 119.7 kDa) was detected using rabbit polyclonal anti-Rv3407 antibody. For each fusion the expected band is indicated by an asterisk. Arrows indicate the position of cleaved fusions or the fusion dimer.
(TIF)

S2 Fig MVATG18377 vaccine is able to trigger a broad IFNγ response specific of Mtb antigens in different mice strains. (**A**) BALB/c, (**B**) C57BL/6 and (**C**) C3H/HeN mice were immunized once with either MVATGN33.1 (light grey) or MVATG18377 (dark grey). Results are shown as the number of IFNγ-producing T cells (spots) per 10^6^ splenocytes following stimulation or not (Medium) with either all peptide pools (P) covering each of the 14 antigens or the irrelevant GLL peptide (Irr). Full bars represent individual mice and hatched bars represent median values of each group. The experimental cut-off value (dotted line) is represented for each mice strain: 51 spots/10^6^ cells for BALB/c, 56 spots/10^6^ cells for C57BL/6 and 72 spots/10^6^ cells for C3H/HeN mice. Results are representative of two independent experiments.
(TIF)

S1 Protocol Western blot analysis and Peptide library. (DOCX)
