# Supplementary figures and images for: A Novel MVA-Based Multiphasic Vaccine for Prevention or Treatment of Tuberculosis Induces Broad and Multifunctional Cell-Mediated Immunity in Mice and Primates
Source: PLoS One. 2015 Nov 24;10(11):e0143552. doi: 10.1371/journal.pone.0143552 (PMC4658014; doi:10.1371/journal.pone.0143552)

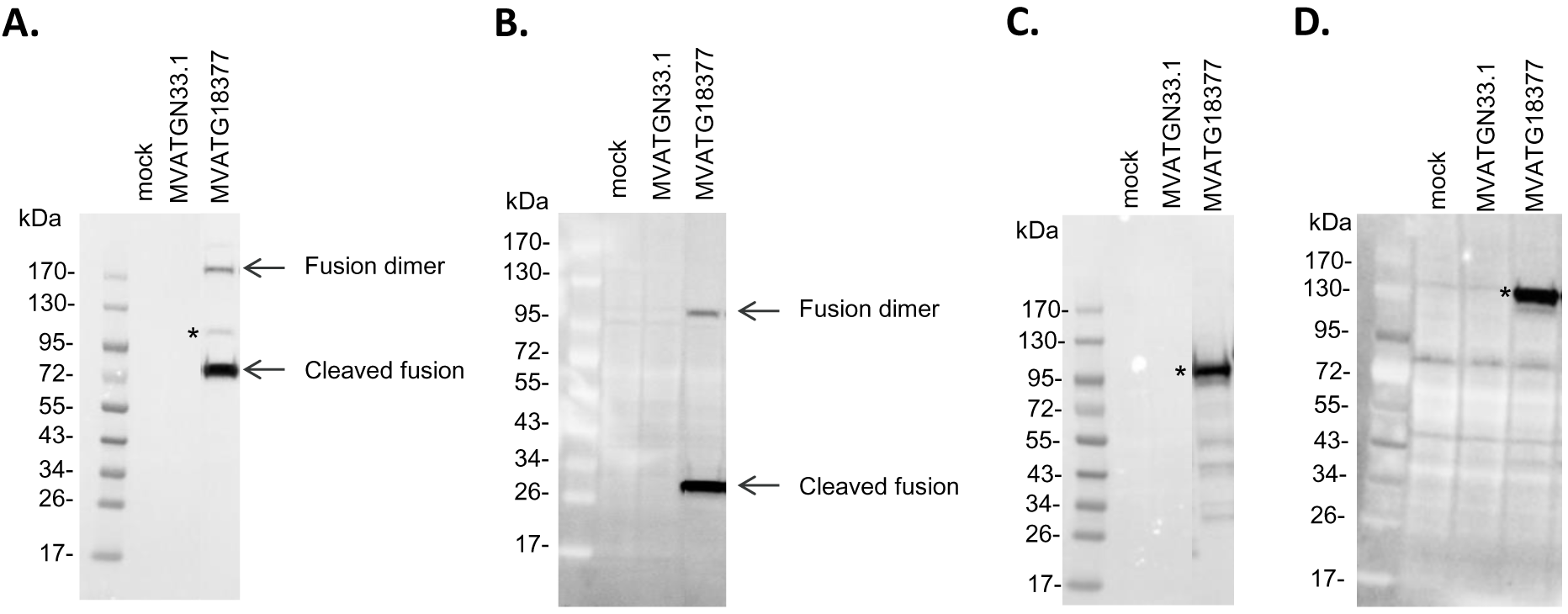

Supplement: S1 Fig — A549 cells were infected or not (mock) with MVATG18377 or MVATGN33.1 and cell extracts analyzed by Western blot. Rv2029-Rv2626-Rv1733-Rv0111 fusion (*, expected molecular weight: 98.4 kDa) was detected using (A) mouse monoclonal anti-Rv2626 26A11 antibody or (B) rabbit polyclonal anti-Rv0111 serum. Major proteolytic products were observed with a Rv2626-specific antibody (around 70.0 kDa) and with Rv0111-specific serum (around 30.0 kDa), suggesting a proteolytic cleavage of this fusion. (C) RpfB-RpfD-Ag85B-TB10.4-ESAT-6 fusion (*, expected molecular weight: 87.0 kDa) was detected using mouse monoclonal anti-ESAT-6 HYB076-08 antibody and (D) Rv0569-Rv1813-Rv3407-Rv3478-Rv1807 fusion (*, expected molecular weight: 119.7 kDa) was detected using rabbit polyclonal anti-Rv3407 antibody. For each fusion the expected band is indicated by an asterisk. Arrows indicate the position of cleaved fusions or the fusion dimer. (TIF) [file pone.0143552.s001.tif]

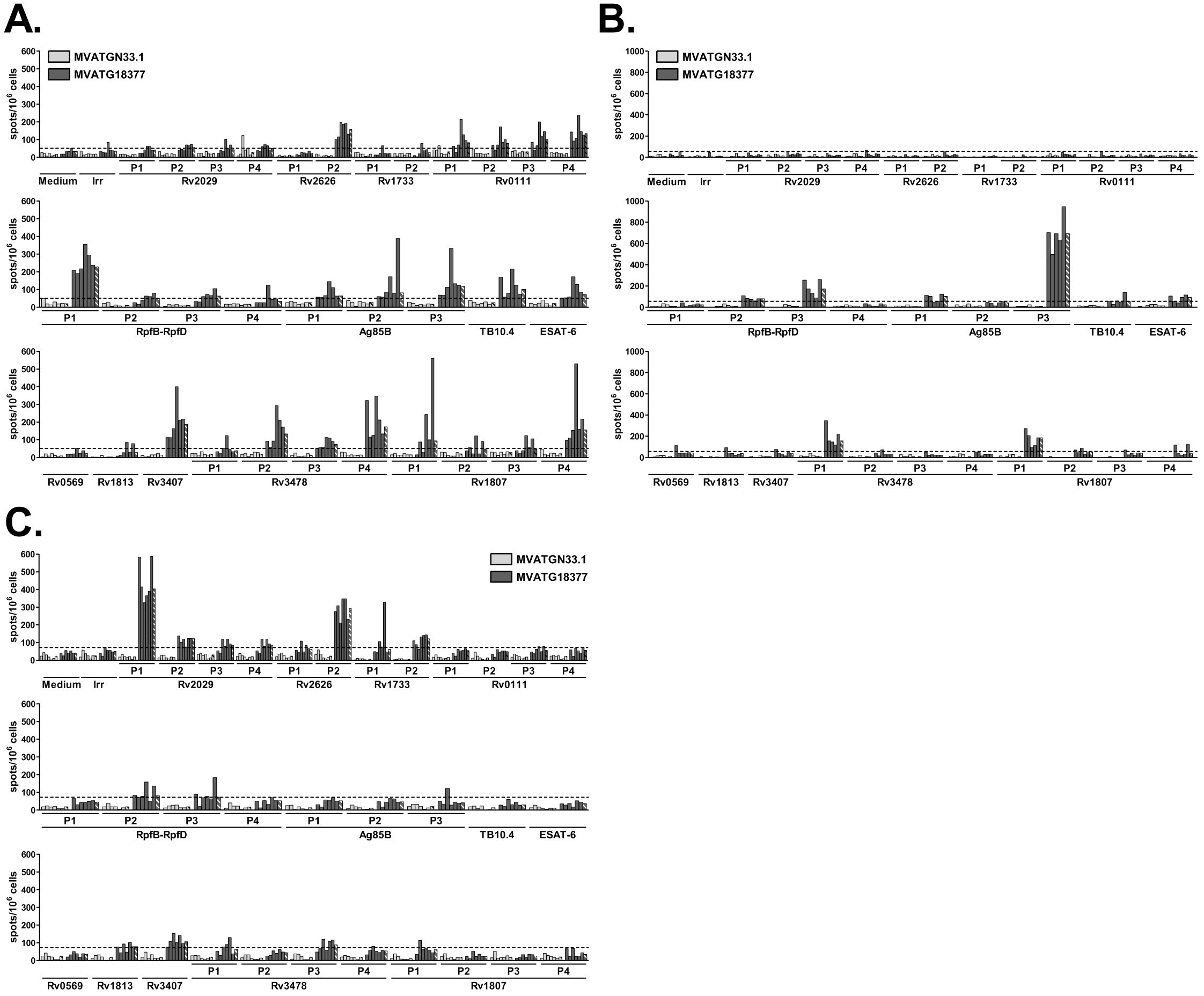

Supplement: S2 Fig — (A) BALB/c, (B) C57BL/6 and (C) C3H/HeN mice were immunized once with either MVATGN33.1 (light grey) or MVATG18377 (dark grey). Results are shown as the number of IFNγ-producing T cells (spots) per 106 splenocytes following stimulation or not (Medium) with either all peptide pools (P) covering each of the 14 antigens or the irrelevant GLL peptide (Irr). Full bars represent individual mice and hatched bars represent median values of each group. The experimental cut-off value (dotted line) is represented for each mice strain: 51 spots/106 cells for BALB/c, 56 spots/106 cells for C57BL/6 and 72 spots/106 cells for C3H/HeN mice. Results are representative of two independent experiments. (TIF) [file pone.0143552.s002.tif]
