# Supplementary material for: A Novel MVA-Based Multiphasic Vaccine for Prevention or Treatment of Tuberculosis Induces Broad and Multifunctional Cell-Mediated Immunity in Mice and Primates
Source: PLoS One. 2015 Nov 24;10(11):e0143552. doi: 10.1371/journal.pone.0143552 (PMC4658014; doi:10.1371/journal.pone.0143552)
Supplement: S1 Protocol — (DOCX) [file pone.0143552.s003.docx]

**S1 Protocol**

**Western-blot analysis**

Monolayers of A549 cells were mock-infected with MVATGN33.1 or infected at a multiplicity of infection of 1 with MVATG18377. MG132 (proteasome inhibitor, 10 µM) was added 30 min post-infection and cells were lysed 24 h post-infection. Infected cells lysate were subjected to SDS-PAGE (4-15 % Criterion^TM^ TGX^TM^ gels, Biorad) and the separated proteins were transferred to PVDF membranes for immunoblotting. The following primary antibodies were used for the detection of mycobacterial fusions: monoclonal antibody for ESAT6 (clone HYB076-08, Santa-Cruz); monoclonal antibody for Rv2626 (clone 26A11, Lifespan-Biosciences) and polyclonal rabbit antibody for Rv3407 and for Rv0111 (generated by Eurogentec; Seraing, Belgium). Goat anti-mouse or anti-rabbit HRP conjugated antibodies (DakoCytomation) were used as secondary antibody and immunocomplexes were detected using an enhanced HRP-luminol chemiluminescence system (Immune-Star Western-C, Biorad).

**Peptide library**

In order to stimulate *ex vivo* splenocytes from immunized mice, we used a peptide library (synthesized by ProImmune using protocol prospector LCMS) constituted by 15-mer peptides overlapping by 11 amino acids (aa). To cover all antigens, 679 peptides were synthesized. For each antigen, pools of peptides were constituted, each pool containing 25 peptides maximum. Therefore, 1 to 4 pools for each respective antigen were needed in order to cover the full length of the given antigens as described below:

- Rv1733 was covered by 2 pools of 18 and 17 peptides. Pool 1: 18 peptides covering Rv1733 residues 62 to 144; Pool 2: 17 peptides covering Rv1733 residues 134 to 210.
- Rv2029 was covered by 4 pools of 19 peptides. Pool 1: 19 peptides covering Rv2029 residues 1 to 87; Pool 2: 19 peptides covering Rv2029 residues 77 to 163; Pool 3: 19 peptides covering Rv2029 residues 153 to 239; Pool 4: 19 peptides covering Rv2029 residues 229 to 314.
- Rv0569 was covered by 1 pool of 20 peptides. Pool 1: 20 covering Rv0569 residues 1 to 88.
- Rv1807 was covered by 4 pools of 25 peptides for the first 3 pools and 22 peptides for the fourth pool. Pool 1: 25 peptides covering Rv1807 residues 1 to 111; Pool 2: 25 peptides covering Rv1807 residues 101 to 211; Pool 3: 25 peptides covering Rv1807 residues 201 to 311; Pool 4: 22 peptides covering Rv1807 residues 301 to 399.
- Rv0111 was covered by 4 pools of 20 peptides for the first 3 pools and 19 peptides for the fourth pool. Pool 1: 20 peptides covering Rv0111 residues 361 to 451; Pool 2: 20 peptides covering Rv0111 residues 441 to 531; Pool 3: 20 peptides covering Rv0111 residues 521 to 611; Pool 4: 19 peptides covering Rv0111 residues 601 to 685.
- RpfB-RpfD was covered by 4 pools of 22 peptides for the first 3 pools and 19 peptides for the fourth pool. Pool 1: 22 peptides covering RpfB residues 30 to 127; Pool 2: 22 peptides covering RpfB residues 117 to 215; Pool 3: 22 peptides covering RpfB residues 205 to 284 and RpfD residues 53 to 71; Pool 4: 19 peptides covering RpfD residues 61 to 146.
- Rv1813 was covered by 1 pool of 25 peptides. Pool 1: 18 peptides covering Rv1813 residues 34 to 143.
- Rv3407 was covered by 1 pool of 22 peptides. Pool 1: 18 peptides covering Rv3407 residues 1 to 99.
- Rv3478 was covered by 4 pools of 24 peptides. Pool 1: 24 peptides covering Rv3478 residues 1 to 107; Pool 2: 24 peptides covering Rv3478 residues 97 to 203; Pool 3: 24 peptides covering Rv3478 residues 193 to 299; Pool 4: 24 peptides covering Rv3478 residues 289 to 393.
- Rv2626 was covered by 2 pools of 17 and 16 peptides. Pool 1: 17 peptides covering Rv2626 residues 1 to 79; Pool 2: 16 peptides covering Rv2626 residues 69 to 143.
- Ag85B was covered by 3 pools of 23 peptides. Pool 1: 23 peptides covering Ag85B residues 39 to 141; Pool 2: 23 peptides covering Ag85B residues 131 to 233; Pool 3: 23 peptides covering Ag85B residues 223 to 325.
- ESAT-6 was covered by 1 pool of 21 peptides. Pool 1: 21 peptides covering ESAT-6 residues 1 to 95.
- TB10.4 was covered by 1 pool of 21 peptides. Pool 1: 21 peptides covering TB10.4 residues 1 to 95.

Individual peptides were resuspended in DMSO at 10 mM in average. Peptides were pooled as described previously. Concentration of each peptide in each pool was 0.4 mM. For *ex vivo* stimulation, peptide pools were used at 1 µM final concentration. Resuspended peptide stocks and pools of peptides were stored at -20 °C.
